# Self-initiated strategies for managing loneliness: insights from two large-scale surveys

**DOI:** 10.3389/fpsyt.2026.1846133

**Published:** 2026-06-12

**Authors:** Wahida Walibhai, Manuela Barreto, Christina Victor, Pamela Qualter

**Affiliations:** 1 Manchester Institute of Education, University of Manchester, Manchester, United Kingdom; 2 Department of Psychology, University of Exeter, Exeter, United Kingdom; 3 Department of Health Sciences, Brunel University London, London, United Kingdom

**Keywords:** asset-based approach, coping, coping strategies, interventions, loneliness, management strategies

## Abstract

**Introduction:**

Most of the research on reducing loneliness has taken a deficit-based approach that focuses on formal interventions rather than an asset-based approach that empowers individuals to manage their own loneliness. There is little understanding of the self-initiated strategies individuals use to manage their loneliness, and a lack of clarity on how perceived effectiveness and use of those strategies differs across sociodemographic characteristics.

**Methods:**

Secondary analysis of two large-scale datasets (BBC Loneliness Experiment and EU Loneliness Survey) was conducted to explore self-initiated loneliness management strategies implemented and perceived to be effective by individuals ages 16 to 99 years who reported frequent loneliness (N = 18354). Respondents selected the strategies they used from a list of 12 pre-defined options in the EU dataset, and the strategies they perceived as effective from a list of 21 options in the BBC dataset. Frequencies of use and perceived effectiveness for each strategy were calculated, and binary logistic regression analyses assessed whether gender, age, income, and geographical region were significant predictors of strategy use and perceived effectiveness.

**Results:**

The most frequently used strategies were seeing friends, family members or other loved ones (31.8%) and taking time for yourself (28.9%). The strategies most frequently perceived as effective were finding activities to distract you when on your own (58.5%) and dedicating time to work, study, or hobbies (52.6%). A strategy used infrequently was contacting a specialized charity (5.3%), and introducing yourself to neighbors (9.5%) was least often perceived as effective. Gender, age, income, and region significantly predicted the use and perceived effectiveness of different strategies.

**Conclusion:**

Individuals experiencing loneliness engage in and perceive as effective various self-initiated, unstructured strategies to manage their loneliness. Future research should consider integrating an asset-based approach that explores the experiences of self-initiated loneliness management, understands the choice of these strategies, and determines their effectiveness to inform future policy and practice.

## 1 Introduction

Loneliness is a subjective and distressing experience that arises when there is a perceived discrepancy between the social relationships individuals desire and those they actually have (1). It is well established that loneliness is prevalent across the life course (2) and associated with adverse mental health, physical health, and socio-economic outcomes (3–5). As a result, loneliness has been increasingly recognized as a global public health priority (6).

Growing attention on loneliness has seen a proliferation of interventions aimed at alleviating loneliness in recent decades, ranging from social skills training and structured group activities to cognitive-behavioral approaches that target maladaptive social cognition (7). Recent meta-analytic evidence supports the general effectiveness of such interventions across different age groups, with small to moderate significant effects in the short term (up to one month) and long term (1–6 months), but raises concerns about the quality of many evaluations (8).

Less attention has been given to how individuals actively manage loneliness on their own. Evidence from across the life course (9) and longitudinal evidence (10) differentiates between temporary and ‘chronic’ loneliness, the latter of which has been characterized by greater duration, frequency or intensity of loneliness (11). This means that for many individuals, loneliness is a transient experience that may arise as an adaptive response to particular life events, and resolves over time (12). While feelings of loneliness might naturally subside for some with time, it is likely that individuals also actively employ strategies to manage their feelings of loneliness. Focusing on how individuals experience and manage this common human experience with agency provides a helpful shift away from stigmatizing narratives about passive victims of a loneliness epidemic or loneliness as an individual deficit (13).

Asset-based approaches to health and wellbeing (14, 15) challenge deficit models that focus on identifying problems and needs within populations and introducing professional interventions. Instead, asset-based approaches recognize the strengths of individuals and communities, and empower them to build upon and use those resources, reducing dependence on professional services. Current approaches to tackling loneliness are largely interventionist and focus on rectifying individual or interpersonal inadequacies (16); in other words, they are often deficit-based. Little research has been conducted to identify the assets of individuals experiencing loneliness and how those behaviors and resources could be built upon to address loneliness and loneliness inequalities.

There is a small body of literature that demonstrates people who report loneliness are not passive recipients of interventions, but, instead, active agents in developing their own coping strategies. Individuals frequently attempt to cope with their loneliness through diverse means such as seeking social interactions, building and altering relationships, self-reflection, engaging with religion, social withdrawal, acceptance, cognitive restructuring, making lifestyle changes, and engaging with hobbies, school and work (17–19). One recent study (20) developed a comprehensive typology of coping strategies used by adults in the United States when they feel lonely through inductive analysis of open-ended survey questions. They examined how commonly those strategies were used, and how effective individuals perceived them to be based on whether they were reported as a successful strategy or one that did not work. Crucially, the authors identified no single coping strategy that was perceived as effective for everyone; each strategy was helpful for some individuals in overcoming their loneliness; the same strategies were also unhelpful for others. This pattern mirrors evidence from formal loneliness interventions, which also shows substantial heterogeneity in effectiveness dependent on context (8).

Since loneliness arises from a range of contexts and experiences, some authors have advocated for tailoring loneliness interventions to address individuals’ preferences and the context of their loneliness experience (21, 22). This requires understanding the role of individual characteristics, including age, gender, socioeconomic status, and region, in how people experience and respond to loneliness. These sociodemographic factors shape individuals’ vulnerability to loneliness, but they also influence the strategies they perceive as accessible and effective. For instance, younger individuals may be more likely to use peer-oriented (23) or digital coping strategies (24), whereas older adults may rely more on individual resilience and positive thinking (25). Gender norms may also influence what people do when they feel lonely, with research suggesting that men in some contexts are less likely to seek emotional support due to prevailing ideals of self-reliance and emotional restraint (26, 27). Additionally, financial constraints may limit participation in social activities or access to formal interventions, particularly in deprived areas where social infrastructure is often underdeveloped (28).

Cultural norms and regional contexts further shape how loneliness is experienced, interpreted, and managed. In collectivist cultures, where social embeddedness and family obligations are emphasized, loneliness may arise due to deviations from social norms and be especially distressing or less openly acknowledged; in individualistic societies, loneliness may arise from weaker familial bonds or social atomization (29–31). While some studies have found higher levels of loneliness in collectivist cultures, others report the opposite, suggesting that cultural values interact in complex ways with personal expectations and social realities (32, 33). Regional differences within countries, such as disparities in investment in social infrastructure or differing levels of community cohesion (34), also play a critical role in shaping loneliness and the perceived utility of available coping strategies. A comprehensive understanding of how individuals manage loneliness must, therefore, account for those dimensions of context and inequality to avoid imposing narrow, culturally biased models of what ‘effective’ support is.

There is a growing emphasis in public health and social policy on identifying effective interventions that are scalable (35, 36). However, loneliness is a complex construct shaped by diverse contexts and individual preferences, meaning there is no “one-size-fits-all” intervention (37). The field currently lacks a clear understanding of what strategies to manage loneliness work best for whom, under what circumstances, and for how long. Therefore, there is insufficient clarity on what parameters interventions should be scaled around. As we have outlined, most existing studies take a deficit-based approach that focuses on formal interventions delivered by practitioners which are difficult and costly to scale; they overlook the everyday, informal, and self-initiated strategies that individuals use to mitigate their loneliness which are likely shaped by people’s experiences, preferences and circumstances. Addressing that gap is crucial in developing an asset-based approach that considers the existing resources of people experiencing loneliness that can be readily mobilized, hence designing interventions that are contextual, scalable and equitable.

The present study shifts the focus from loneliness as an outcome to be explained to loneliness as a condition people often actively seek to change. While previous research has examined how frequently different strategies are used to address loneliness and their perceived effectiveness in the United States (20), the current study explores the frequency and perceived effectiveness of strategy use across a global context. Importantly, the current study builds on previous work by examining how the use and perceived effectiveness of strategies vary across sociodemographic characteristics.

Drawing on large-scale datasets from the EU Loneliness Survey and the BBC Loneliness Experiment, we examine the frequency of use of strategies to manage loneliness by individuals reporting loneliness, the perceived effectiveness of those strategies, and how use and perceived effectiveness differ by age, gender, income, and region. By analyzing both self-initiated coping behaviors and their sociodemographic patterning, the current study expands understanding of how individual resources, preferences, and accessibility may impact coping with loneliness, informing the development of an asset-based approach to loneliness which is more nuanced, equitable, and context-sensitive.

## 2 Methods and materials

### 2.1 Participants and procedure

This study is a secondary analysis of data from two independent large datasets.

The EU Loneliness survey (38) was an online questionnaire conducted in November and December 2022 with two samples: EU4 and EU27. The EU4 sample collected data from participants ages 16–91 years from four EU member states (France, Italy, Poland and Sweden). Approximately 1000 participants from each country took part, resulting in 4029 respondents. The EU27 sample consisted of 25646 participants ages 16 to 91 years from all 27 EU member states (including the member states that were part of EU4), who completed a slightly longer survey. Our study used data from the 3507 participants across both EU samples who indicated that they felt lonely most or all of the time. For binary logistic regression analyses, data from 3486 participants were analyzed, excluding those with missing gender, age or region data.

The BBC Loneliness Experiment (39) was an online survey collected from February to May 2018 that included 55060 participants ages 16–99 years from 237 countries, islands, and territories. The current study analyzed data from the 14847 participants who reported experiencing loneliness most or all of the time. For binary logistic regression analyses involving the BBC dataset, participants who had missing gender, age or income data were excluded, resulting in 14364 participants.

In total, we analyzed data from 18354 participants across both datasets.

### 2.2 Measures

#### 2.2.1 Loneliness

In the EU dataset, a single loneliness item “How much of the time, during the past 4 weeks, have you been feeling lonely?” was measured on a 5-point scale (1 = All of the time, 2 = Most of the time, 3 = Some of the time, 4 = A little of the time, 5 = None of the time). Participants who scored 1 or 2 were classified as ‘lonely’ in our analysis. In the BBC dataset, participants completed a single-item measure of loneliness “How often do you feel lonely?”. Participants rated this item on a 5-point scale (1 = Never, 5 = Always). Participants who scored 4 or 5 were classified as ‘lonely’ for our analysis. While both datasets included other measures of loneliness, the direct, self-reported, single-item measure of loneliness frequency was used in the current study because it has previously demonstrated adequate test-retest reliability and convergent validity with other loneliness measures, indicating adequate construct validity (40).

#### 2.2.2 Use of strategies

The EU dataset asked “In the last 12 months, have you done any of the following to feel less lonely?” and provided 16 answer options. This question was used as a measure of the use of different loneliness coping strategies. Two of the answer options were excluded from our analysis because they did not convey any meaningful information (Don’t know, and Prefer not to say). One of the options (Other, not listed above) did not specify a strategy, and one of the options (None of the above) conveyed that the individual did not utilize any of the listed loneliness management strategies. Those options were retained in the analysis because they still conveyed important information about whether individuals used any strategies. The final 12 answer options used in our analyses included doing sports with others and seeking professional help from a therapist (see **Table 1** for all strategies in the EU dataset).

**Table 1:** Number and percentage of lonely participants who utilized each strategy in the EU dataset.

Strategy	Count (%)
Seeing friends, family members, or other loved ones	1116 (31.82%)
Doing sports alone	722 (20.59%)
Doing sports with others	380 (10.84%)
Looked for self-help from books or online or called a support hotline	464 (13.23%)
Sought professional help by a therapist	456 (13.00%)
Contacted a specialized charity, association or non-governmental organization	184 (5.25%)
Contacted a church or religious organization	209 (5.96%)
Joined a club or group	321 (9.15%)
Volunteered	257 (7.33%)
Used more social media	1002 (28.57%)
Took time for myself	1015 (28.94%)
Wanted to do something but did not know what to do	633 (18.05%)
Other, not listed above	123 (3.51%)
None of the above	295 (8.41%)
	Total = 3507

#### 2.2.3 Perceived effectiveness of strategies

The BBC dataset included the following question: “Please think about which of these possible solutions to loneliness you, or others you know, have found to be effective at reducing feelings of loneliness. Please select all that apply.” 21 potential loneliness management strategy options were provided, such as joining a club and looking for a new job (see **Table 2** for all strategies in the BBC dataset). We used this question as a measure of whether each strategy was perceived as effective in managing loneliness.

**Table 2:** Number and percentage of lonely participants who perceived each strategy in the BBC dataset to be effective.

Strategy	Count (%)
Join a club	6013 (40.50%)
Find activities to distract you when on your own	8692 (58.54%)
Dedicate time to work, study or hobbies	7810 (52.60%)
Look for a new job	1903 (12.82%)
Introduce yourself to neighbors	1415 (9.53%)
Move to a new area	1490 (10.04%)
Re-engage with your church, mosque or equivalent	2036 (13.71%)
Tell someone	3450 (23.24%)
Talk to family and friends about your feelings	4331 (29.17%)
Invite people to be friends without fearing rejection	2862 (19.28%)
Look for the good in everyone you meet	4023 (27.10%)
Start a conversation with anyone you interact with	4826 (32.50%)
Seek counselling	4274 (28.79%)
Find new non social activities and pastimes	3082 (20.76%)
Find new friends	3126 (21.05%)
Use the internet for support	3498 (23.56%)
Wait for the feeling to pass	4843 (32.62%)
Give yourself time to think about why	3496 (23.55%)
Don’t know what to do	4007 (26.99%)
Find new social activities and pastimes	4936 (33.25%)
Change my thinking to be more positive	5494 (37.00%)
	Total = 14847

#### 2.2.4 Predictors

Participants in both datasets provided demographic information that was analyzed to explore whether they predicted use of each loneliness coping strategy (in the EU dataset) or the perceived effectiveness of coping strategies (in the BBC dataset). *Gender* (male, female, other) was measured in both datasets, as was *Age* which we categorized into three groups based on World Health Organization definitions of young people (41) and older people (42) [young adults (16–24 years), adults (25–59 years) and older adults (60+ years)]. Broad age groups were selected as youth and older adults are considered higher risk age groups for experiencing loneliness, while loneliness prevalence remains relatively stable across adulthood (43).

We also created a predictor variable in the EU dataset called *Region*, with four categories based on participants’ countries of birth (Western, Northern, Southern, or Central and Eastern Europe). The four regions were categorized according to EuroVoc (44), a thesaurus maintained by the Publications Office of the European Union (see **Supplementary Table 1** for region classifications for each of the 27 EU countries). An additional categorical predictor found only in the BBC dataset was *Income* which was assessed with the question “How well do you feel your needs are met by the financial resources you have (i.e. money)?” (very well, fairly well, poorly).

### 2.3 Statistical analysis

First, we described the frequency of use of each loneliness coping strategy by participants classified as lonely, and how many participants perceived each strategy to be effective. All predictor variables (age category, gender, region, and income) were categorical, and the percentage of participants in each category were calculated in both datasets. Next, multicollinearity between predictor variables was assessed through Spearman’s rank-order correlations between age category, gender and region for the EU dataset and age category, gender and income in the BBC sample.

To determine the sociodemographic patterning of utilizing different loneliness coping strategies, we conducted binary logistic regression analyses with each strategy in the EU dataset as the outcome and gender, age category, and region as predictors of use. 14 logistic regressions were conducted for the 14 strategy options. 21 further binary logistic regressions analyzed the prediction of age category, gender and income for perceiving strategies in the BBC dataset as effective. For each model, the regression coefficients (*B*), standard errors, odds ratios, 95% confidence intervals, and *p*-values are reported. Nagelkerke R^2^ values are reported as an estimate of the proportion of variance in the use or perceived effectiveness of each strategy that was explained by the predictor variables. Due to the large number of regressions conducted (35 in total), a conservative significance level of *p* <.001 was set. All analyses were conducted using SPSS version 29.0.

## 3 Results

### 3.1 Descriptive statistics

In the EU dataset, 3507 from 28554 participants (12.2%) indicated that they felt lonely most or all of the time during the last 4 weeks. 44626 participants from the BBC dataset completed our selected loneliness measure and, of those, 14847 (33.3%) felt lonely mostly or very often. Counts and percentage distributions of lonely participants’ age category, gender, region (for EU participants), and income (for BBC participants) are presented in **Table 3**. Distributions of gender and age category appeared similar across datasets.

**Table 3:** Number and percentage of lonely participants in each predictor variable category. .

Variable		N	Percentage
EU participants (N = 3486)			
Age Category	Adults	2561	73.47
	Young adults	555	15.92
	Older adults	370	10.61
Gender	Male	1554	44.58
	Female	1901	54.53
	Other	31	0.89
Region	Western	949	27.22
	Northern	835	23.95
	Southern	712	20.42
	Central and Eastern	990	28.40
BBC participants (N = 14364)			
Age Category	Adults	9304	64.77
	Young adults	1275	8.88
	Older adults	3785	26.35
Gender	Male	5141	35.79
	Female	9129	63.55
	Other	94	0.65
Income	Poorly	4015	27.95
	Fairly	7161	49.85
	Very	3188	22.19

### 3.2 Use of strategies

Findings show a range of strategies were used by participants in the EU loneliness survey to manage feelings of loneliness. A summary of percentage distributions of the number of strategies used by participants classified as lonely are displayed in **Table 4** (a more detailed breakdown can be found in **Supplementary Table 2**). The number of strategies used ranged from 0 to 11 out of a maximum of 12 strategies. The majority of individuals used at least one loneliness strategy, while 30.3% of participants reported not using any strategies. The mode number of strategies used was 0.

**Table 4:** Number of strategies used and perceived to be effective by lonely participants in the EU (N = 3507) and BBC (N = 14847) datasets respectively.

Number of strategies	Used (% of EU participants)	Perceived as effective (% of BBC participants)
0	30.34	13.31
1-3	53.07	22.48
4-6	14.95	25.41
7-9	1.48	10.66
10+	0.18	18.15

**Figure 1:**
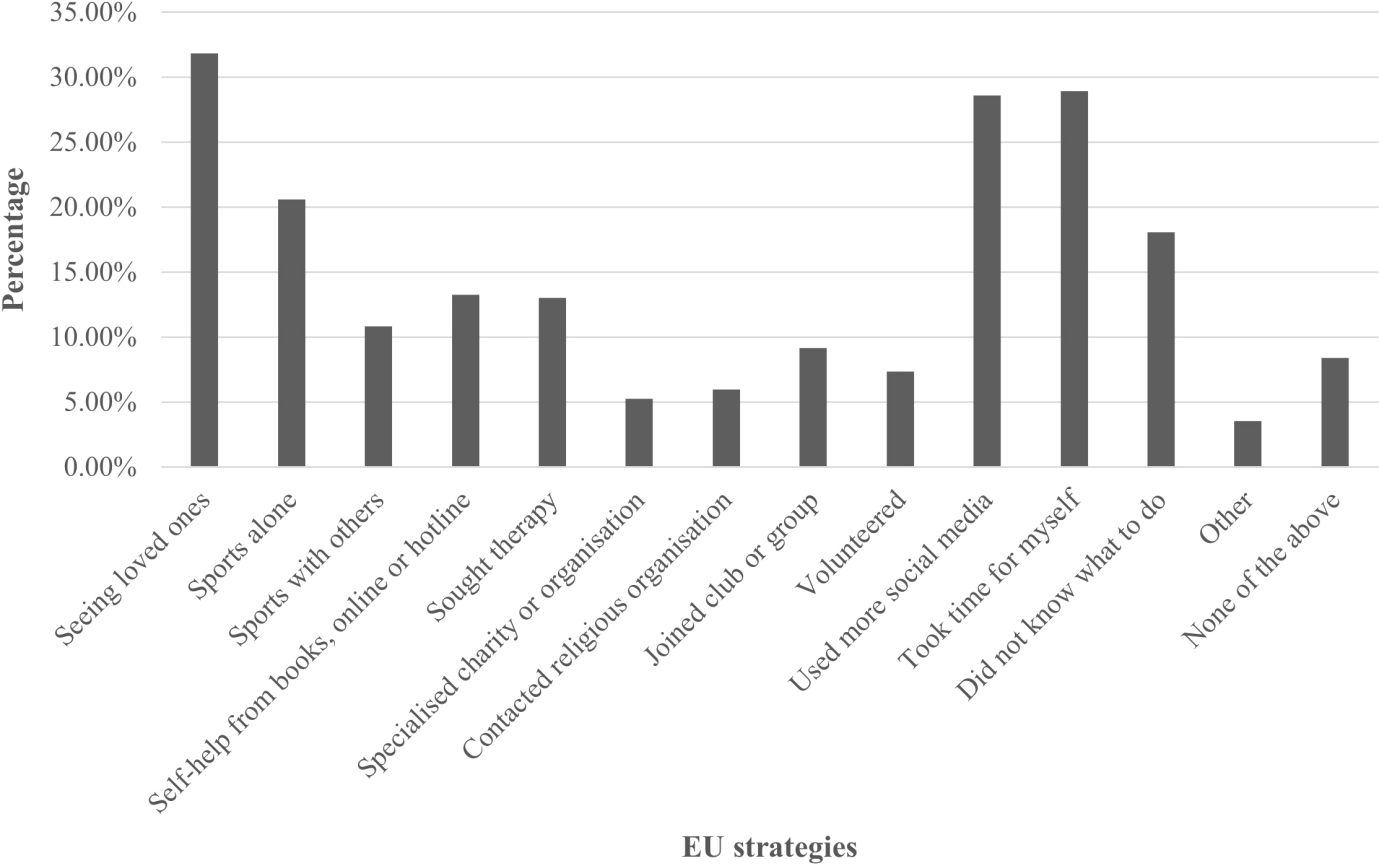
Percentage of lonely participants who utilized each strategy in the EU dataset.

**Table 1** and **Figure 1** summarize the number and percentage of lonely participants that used each loneliness management strategy in the EU dataset. Use of strategies ranged between 3.51% (other solution, not listed) and 31.82% (seeing friends, family and loved ones), with the least used named strategy being contacting a specialized charity, association, or non-governmental organization (5.25%).

### 3.3 Perceived effectiveness of strategies

In the BBC dataset, a range of strategies to manage loneliness were perceived as effective by participants we classified as lonely. **Table 4** summarizes the percentage distribution of the number of strategies participants perceived as effective (see **Supplementary Table 3** for a more detailed breakdown). The number of strategies perceived as effective ranged from 0 to the maximum of 21 strategies, with only 13.3% of lonely participants not perceiving any strategies to be effective. The mode number of strategies perceived as effective was 3 (17.0%).

**Figure 2:**
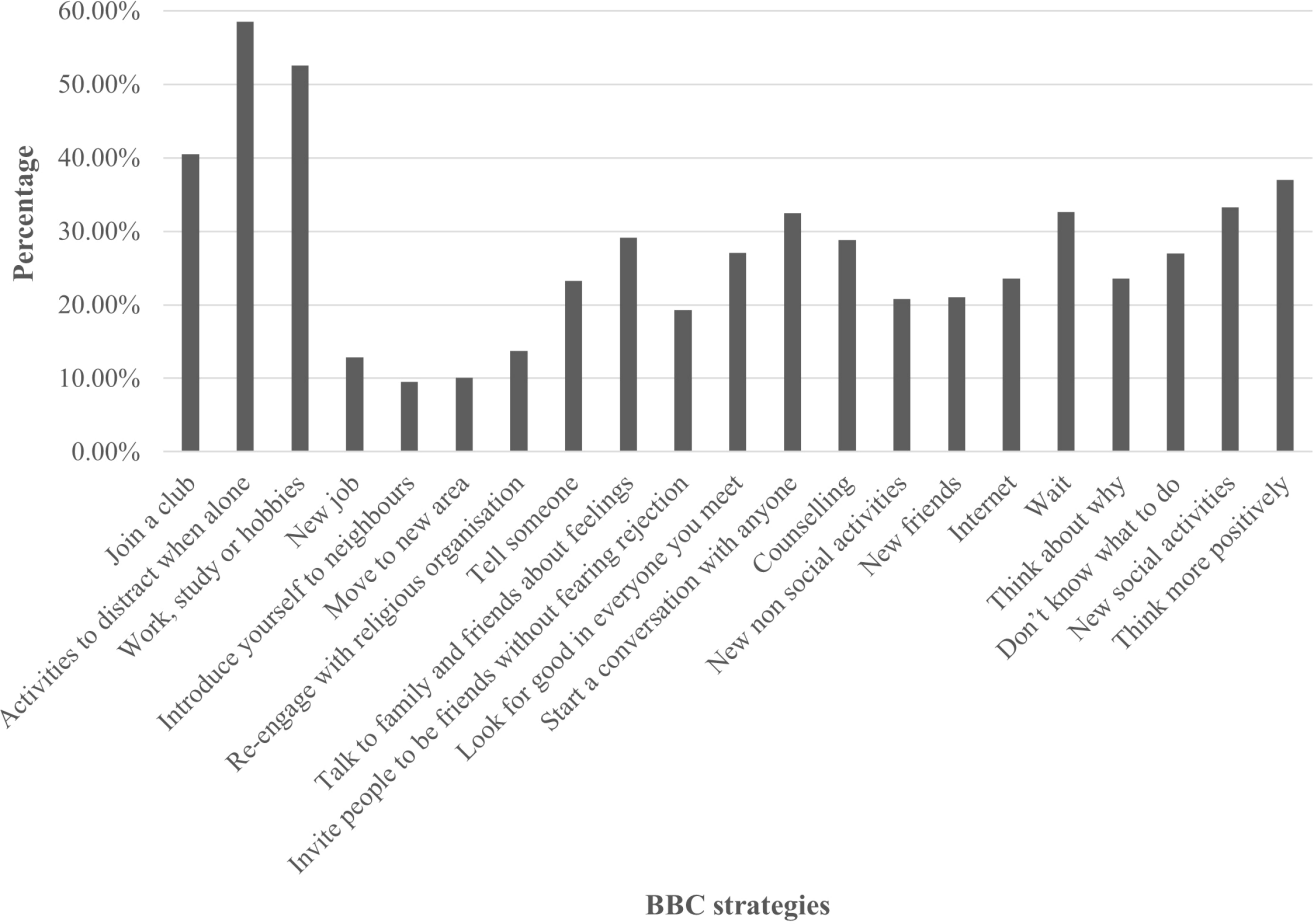
Percentage of lonely participants who perceived each strategy as effective in the BBC dataset.

**Table 2** and **Figure 2** display the number and percentage of participants classified as lonely in the BBC dataset who perceived each loneliness management strategy as effective. Results ranged from 9.53% of participants perceiving introducing yourself to neighbors to be an effective strategy, to 58.52% perceiving finding activities to distract you when on your own as effective.

### 3.4 Logistic regression models

Prior to conducting the logistic regressions, Spearman’s rank-order correlations were calculated to assess potential multicollinearity between all predictor variables. No predictor pairs resulted in a correlation coefficient of ρ >.70 or ρ < -.70, indicating acceptable independence among predictors.

#### 3.4.1 How do sociodemographic characteristics predict strategies used to manage loneliness in the EU dataset?

Binary logistic regression models examined the associations between gender, age category and region, and utilization of each of the 14 loneliness management strategy options by individuals who reported that they were lonely most or all of the time in the EU dataset and did not have missing relevant data (N = 3486). Regression coefficients, standard errors, odds ratios (OR), 95% confidence intervals (CIs), *p*-values, and Nagelkerke R^2^ values are reported in **Supplementary Table 4**. Here, the results are presented with a focus on which strategies were predicted by each sociodemographic characteristic, but **Supplementary Table 4** displays the full regression models for each strategy option.

Gender was a significant predictor of doing sports with others, contacting a specialized charity, contacting a religious organization, joining a club, and wanting to do something but not knowing what to do. Females were significantly less likely than males to engage in all of those strategies, except for not knowing what to do, which they were significantly more likely to select.

Age category significantly predicted seeing loved ones, doing sports alone or with others, seeking therapy, using more social media, and taking time for oneself, with young adults being more likely to utilize all of those compared to adults, and older adults being less likely than adults to seek professional help by a therapist. Age category was also a significant predictor of not using any management strategies, although young adults and older adults did not significantly differ in doing so from adults, suggesting that the effect of age may have been distributed across age groups rather than driven by one significantly different group.

Additionally, region was a significant predictor of seeing loved ones, contacting a specialized charity, joining a club, and using more social media. Respondents in Northern (e.g. Denmark), Southern (e.g. Portugal) and Central and Eastern (e.g. Poland) Europe had significantly greater odds of seeing loved ones and significantly fewer odds of joining a club compared to Western Europeans (e.g. France). Participants in Northern and Southern Europe were also significantly more likely to use social media than Western Europeans. Furthermore, Central and Eastern Europeans were significantly less likely to contact a specialized charity than those in Western Europe.

#### 3.4.2 How do sociodemographic characteristics predict the perceived effectiveness of strategies to manage loneliness in the BBC dataset?

Further binary logistic regression analyses examined the association between each of the 21 strategies from the BBC dataset (N = 14364), and the predictors of gender, age category, and income. **Supplementary Table 5** displays the results.

Gender significantly predicted the perceived effectiveness of finding activities to distract you when alone, dedicating time to work, study or hobbies, introducing yourself to neighbors, re-engaging with your place of worship, telling someone, talking to family and friends about your feelings, inviting people to be friends without fearing rejection, looking for the good in everyone you meet, starting a conversation with anyone you interact with, seeking counselling, using the internet for support, thinking about why you are lonely, not knowing what to do, finding new social activities, and thinking more positively. For all of those strategies, females were significantly more likely than males to perceive them as effective, except that females were significantly less likely to not know what to do. Participants in the ‘other gender’ category were also significantly more likely than males to perceive using the internet for support as effective.

Age category was a significant predictor of most loneliness management strategies. Young adults were significantly more likely than adults to perceive various strategies as effective: telling someone, talking to family and friends, inviting people to be friends without fearing rejection, finding new friends, using the internet for support, giving themselves time to think about why, and they were more likely to not know what to do. Young adults were significantly less likely than adults to perceive starting a conversation with anyone they interact with as effective. Older adults had significantly greater odds of perceiving as effective the strategies of joining a club, finding activities to distract them when alone, introducing themselves to neighbors, looking for the good in everyone they meet and starting a conversation with anyone they interact with compared to adults. Older adults had significantly fewer odds of perceiving as effective: looking for a new job, telling someone, talking to family and friends about their feelings, inviting people to be friends, seeking counselling, giving themselves time to think about why they’re lonely, and they were less likely to not know what to do. Age category also significantly predicted finding new non-social activities, but the direction of this prediction was unclear as young adults and older adults did not significantly differ from adults in the perceived effectiveness of this strategy.

Finally, income was a significant predictor of multiple strategies. People whose income met their needs fairly or very well were significantly more likely to perceive joining a club and finding new social activities as effective than those whose needs were met poorly, and significantly less likely to perceive looking for a new job, moving to a new area, seeking counselling, and using the internet for support as effective. They were also less likely to not know what to do. Moreover, those whose needs were met very well were significantly more likely to perceive inviting people to be friends without fearing rejection as effective than those whose needs were met poorly by their income.

#### 3.4.3 Comparing the sociodemographic patterning of strategies used with those perceived as effective

Four strategies were found in both datasets: seeking therapy/counselling, contacting or re-engaging with a religious organization, joining a club, and not knowing what to do. Other strategies were similar, but did not map onto each other directly. For example, seeing friends, family or loved ones was similar to, but not the same as, talking to family and friends about one’s feelings.

Across both datasets, older adults were both less likely to seek therapy/counselling than adults and less likely to perceive it as effective. While young adults in the EU dataset were additionally more likely to seek therapy than adults, the perceived effectiveness of this strategy did not differ between these two age groups in the BBC dataset. Females were more likely than males to perceive counselling as effective in the BBC dataset, but gender was not a significant predictor of the use of that strategy in the EU dataset. For joining a club, age category was not a significant predictor of use in the EU dataset, but older adults in the BBC dataset were more likely to perceive joining a club as effective than adults. Although gender did not significantly predict perceived effectiveness of joining a club in the BBC dataset, females were less likely than males to join a club in the EU dataset.

Interestingly, gender was the only significant predictor of contacting or re-engaging with a religious organization in both datasets, but the direction of prediction was exactly opposite between datasets: females were less likely than males to use this strategy in the EU dataset, but more likely than males to perceive it as effective in the BBC dataset. The direction of prediction of the gender variable also contrasted for not knowing what to do as females were less likely to not know what to do to manage their loneliness than males in the BBC dataset, whereas they were more likely to not know what to do in the EU dataset. Age was an additional predictor of this variable in the BBC dataset only, with young adults being more likely and older adults being less likely to not know what to do to manage their loneliness than adults.

## 4 Discussion

This study investigated the self-initiated strategies that individuals reporting loneliness use to cope with their loneliness and those they perceive as effective, drawing on large-scale datasets from the BBC Loneliness Experiment and the EU Loneliness Survey. The primary goal was to understand which strategies are most used to manage loneliness, which strategies are most often perceived as effective, and how utilization and perceived effectiveness vary by sociodemographic factors such as age, gender, region, and income. We found that individuals reporting loneliness actively engage in diverse, self-initiated strategies to manage their loneliness, perceive many different strategies as effective, and the strategies used and perceived as effective are to some extent shaped by sociodemographic and contextual factors. We argue that loneliness interventions must move beyond formal, one-size-fits-all models and towards integrating an asset-based approach that recognizes the informal, personalized, cost-effective approaches people already use, mobilizing those existing resources to design scalable, context-sensitive interventions.

### 4.1 The diversity of self-initiated strategies for managing loneliness

Most participants in the EU dataset used at least one self-initiated loneliness management strategy, and around half of participants used multiple strategies. Furthermore, most BBC participants perceived multiple strategies as effective. In fact, around 18% of participants selected 10 or more strategies they perceived as effective in managing loneliness. Those findings support the argument that informal, everyday responses to loneliness are widespread and varied, and that many individuals do not rely on, or indeed have access to, formal interventions. Rather than imposing reliance on professional help, we argue that many individuals experiencing loneliness could benefit from an asset-based approach (14) that builds upon the accessibility of existing self-initiated strategies to manage loneliness.

We found substantial variability in the types of strategies people used to manage experiences of loneliness. The most used strategy was seeing friends, family or other loved ones, which is consistent with previous research in which investing in existing relationships was a frequently reported coping strategy for loneliness (20). Contacting a specialized charity, association, or non-governmental organization was the most infrequently used loneliness management strategy, and this is not a strategy that has been reported in previous literature.

There was also substantial variability in the types of strategies perceived as effective in managing loneliness. The strategy most often perceived as effective was finding activities to distract you when on your own. In contrast, Ray and Rushing (20) reported mixed results on the perceived effectiveness of distractions for coping with loneliness, although they did convey that it was a frequently used strategy. It may seem counterintuitive for a solitary strategy to be perceived as effective for reducing loneliness, but distracting activities can prevent rumination and negative thinking which exacerbate feelings of loneliness (45), and give individuals a sense of autonomy to decide what to do with their solitude, which can make spending time alone a pleasant rather than distressing experience (46). Introducing yourself to neighbors was least often perceived as effective. This falls under the strategy of forming new relationships which was reported in previous literature as commonly used, but with mixed perceived effectiveness (20). Taken together, this suggests that the relationships people seek to form when they are lonely are usually not with neighbors, but instead through clubs, social activities, and spontaneous conversation, which were all more often perceived as effective in the BBC dataset. This is consistent with evidence suggesting a decrease in social ties with neighbors over the last few decades, particularly among young people (47). Furthermore, it confirms that loneliness is not just driven by the desire for more relationships, but that the quality of those new relationships is also important (1, 48).

A key contribution of this study lies in the finding that the use of strategies to manage loneliness is significantly related to individuals’ sociodemographic characteristics. Age was a robust predictor: younger adults were more likely to see loved ones and use social media, while older adults less often reported seeking professional support through therapy and counselling. Such findings align with prior literature suggesting that younger people are more attuned to online help-seeking (49) and emotional support options (50, 51), whereas older adults may lean towards self-management strategies rather than formal psychological interventions (52). These results may in part reflect preferences, but also what options are available to different age groups. For instance, young adults may be more able see loved ones when they are lonely due to greater proximity or contact with family and friends compared to older adults. Furthermore, the findings may reflect differences in what each age group think will be effective in reducing loneliness. Indeed, age was a predictor of perceiving talking to friends and family about your feelings and seeking counselling to be effective for managing loneliness, with the direction of those predictions aligned with the use of the strategies. Importantly, those patterns indicate that interventions aiming to reduce loneliness must consider age-specific preferences, perceived effectiveness, capabilities, and resources.

Gender also played a significant role in shaping loneliness responses. Men were more likely to report using various strategies including doing sports with others, joining clubs, and contacting formal support organizations, and less likely to not know what to do when they were lonely. Nevertheless, the use and perceived effectiveness of strategies appear to diverge when taking gender as a predictor. It was women who were more likely to perceive a wide range of strategies as effective, such as seeking counselling, joining clubs, using the internet for support, and talking about their feelings. Thus, while men employed a greater variety of strategies to manage loneliness in the EU dataset, men in the BBC dataset perceived fewer strategies to be effective. This may suggest that while men are proactive in managing their loneliness, they do not feel that their self-initiated strategies are sufficient to reduce their loneliness. The strategies they frequently engage in like taking part in sports and clubs may provide social contact, but may not address feelings of loneliness based on the quality of social relationships. Existing research highlights gendered norms around emotional disclosure and help-seeking (53, 54) which may create barriers around addressing the quality of relationships. Overall, these results emphasize the limitations of self-initiated strategies and indicate that formal interventions that are responsive to gendered barriers and facilitators, as well as the root causes of loneliness, may be required for some.

The results further underline the importance of contextual and cultural factors. In the EU dataset, regional differences significantly predicted utilization of certain strategies. For example, individuals from Northern, Southern, and Central and Eastern Europe were more likely to report seeing loved ones and less likely to join clubs. Those findings point to differing levels of social infrastructure, community cohesion, and cultural norms around loneliness across European regions. Income was also a significant predictor of the perceived effectiveness of loneliness management strategies. People who reported their financial needs being met “very well” were more likely to perceive joining clubs, inviting others to be friends, and engaging in new social activities as effective in managing loneliness. That finding highlights the material constraints that shape individuals’ ability to act on their desire for social connection.

### 4.2 Implication of findings

Our findings support the central proposition of this study: that people reporting loneliness exhibit agency in their efforts to cope with loneliness, and that their efforts are shaped by sociodemographic and contextual factors. Our findings also challenge the one-size-fits-all approach often implicit in current loneliness interventions, particularly those emphasizing professionalized or formal routes to support (8). While formal interventions (such as therapy or support groups) are important where self-initiated strategies are perceived as ineffective, particularly in improving the quality of relationships, our findings suggest that informal strategies, like introspection, self-care, social initiation, and peer-based connection, are widely used and may better align with some people’s preferences and capacities. As such, we advocate for the integration of an asset-based approach to tackling loneliness to design and research the effectiveness of interventions which align with people’s resources, experiences and preferences.

From a policy perspective, the relatively frequent use and perceived effectiveness of strategies such as sports, clubs, social activities and seeking new relationships highlight the importance of investing in social infrastructure to support individuals in utilizing self-initiated coping strategies. The variation in use and perceived effectiveness of social activities and clubs by region and income may indicate differential access to social spaces, and investment in low-cost, community-based social infrastructure could reduce inequalities in access to such coping strategies. Moreover, our findings suggest that the perceived effectiveness of strategies to tackle loneliness may vary across sociodemographic groups. This could have implications for formal interventions which would benefit from tailoring to age and gender, and potentially regional context, acknowledging that what works for one group may not resonate, or even be accessible, to another. For example, psychological interventions that show effectiveness for younger cohorts should be accompanied by consideration of potential barriers to access or uptake if adapting to other age groups such as older adults.

### 4.3 Limitations and future research

Our classifications of participants as lonely were based on single loneliness items with different temporal framing. In the EU dataset, the item asked about how lonely participants felt in the last 4 weeks, whereas there was no time frame specified in the BBC item. Thus, participants classified as lonely in the EU dataset experienced frequent loneliness recently, whereas participants classified in the BBC dataset experienced frequent loneliness in general, with unknown recency. Although frequency and recency are not the only factors that differentiate ‘chronic’ and temporary loneliness- it is important to consider duration and intensity too (11)- it may be the case that some EU participants were experiencing a recent episode of temporary loneliness, or that some EU participants who experienced ‘chronic’ loneliness were excluded as they had not felt lonely within the past 4 weeks. Complementary and divergent findings in the two samples should, therefore, be considered in light of this differential temporal framing as the datasets may be capturing different experiences of loneliness.

We used two large datasets in our analysis as they provided complementary information on the use of different loneliness management strategies and their perceived effectiveness, which one dataset alone did not provide. However, it should be emphasized that each sample was taken from different global contexts and timepoints: the EU dataset was sampled from the 27 EU member states in 2022, while the BBC dataset recruited participants globally in 2018, including a large proportion of participants from the UK. Geography and pre- and post-COVID-19 pandemic timing may have influenced the accessibility and perceived effectiveness of loneliness management strategies.

Finally, although part of our sample (BBC dataset) focused on strategies perceived to be effective, this may not reflect the actual effectiveness of those strategies in reducing loneliness. Since the experience of loneliness is subjective, which is also reflected in the self-reported measurement of the construct (40), the current study’s exploration of the perceptions of individuals experiencing loneliness in the ability of different strategies to reduce loneliness is still a valuable contribution. Nevertheless, we reiterate Ray and Rushing’s (20) suggestion that future research should investigate the effectiveness of self-initiated coping strategies through longitudinal assessment of loneliness scores. Future research in this area should also consider how sociodemographic characteristics influence the effectiveness of such strategies. Further qualitative work would complement such large-scale analysis by exploring the subjective experiences behind strategy use and effectiveness, including barriers to uptake and emotional outcomes. For example, in-depth qualitative exploration may provide further insight into how taking time for oneself, a frequently used strategy which initially seems counterintuitive due to its solitary nature, may address loneliness, as well as how the causes of loneliness shape the selection and effectiveness of coping strategies.

### 4.4 Conclusion

Individuals who reported loneliness used diverse strategies to manage their experience. Strategies included informal, self-initiated responses that were both self-focused and social in nature; most popularly, seeing loved ones. A wide range of strategies were also perceived as effective in managing loneliness, including finding distracting activities when alone. Strategies used to address loneliness and those perceived as effective varied across sociodemographic characteristics including gender, age, geographic region, and income. Future research should assess the actual effectiveness of self-initiated loneliness management strategies and loneliness intervention research should consider shifting towards an asset-based approach that builds interventions upon what is most accessible and preferred across different sociodemographic characteristics. Policymakers should facilitate equitable access to informal interpersonal loneliness management strategies by investing in low-cost access to third places.

## Data Availability

Publicly available datasets were analyzed in this study. This data can be found here: https://osf.io/pw74v/overview https://data.jrc.ec.europa.eu/dataset/82e60986-9987-4610-ab4a-84f0f5a9193b.
