# Supplementary material for: Self-initiated strategies for managing loneliness: insights from two large-scale surveys
Source: Front Psychiatry. 2026 Jun 12;17:1846133. doi: 10.3389/fpsyt.2026.1846133 (PMC13303519; doi:10.3389/fpsyt.2026.1846133)
Supplement: Supplementary file 1 [file Supplementaryfile1.docx]

***Supplementary Material***

**Supplementary Table 1**. Classifications of EU countries into European regions according to EuroVoc.

| **European region** | **Countries** |
| --- | --- |
| Western | Austria |
|  | Belgium |
|  | France |
|  | Germany |
|  | Ireland |
|  | Luxembourg |
|  | Netherlands |
| Northern | Denmark |
|  | Estonia |
|  | Finland |
|  | Latvia |
|  | Lithuania |
|  | Sweden |
| Southern | Cyprus |
|  | Greece |
|  | Italy |
|  | Malta |
|  | Portugal |
|  | Spain |
| Central and Eastern | Bulgaria |
|  | Croatia |
|  | Czechia |
|  | Hungary |
|  | Poland |
|  | Romania |
|  | Slovakia |
|  | Slovenia |

**Supplementary Table 2.** Number of strategies utilized by lonely participants in the EU dataset.

| **Number of strategies** | **EU** | |
| --- | --- | --- |
|  | **N** | **%** |
| 0 | 1064 | 30.34 |
| 1 | 805 | 22.95 |
| 2 | 555 | 15.83 |
| 3 | 501 | 14.29 |
| 4 | 305 | 8.70 |
| 5 | 147 | 4.19 |
| 6 | 72 | 2.05 |
| 7 | 40 | 1.14 |
| 8 | 7 | 0.20 |
| 9 | 5 | 0.14 |
| 10 | 3 | 0.09 |
| 11 | 3 | 0.09 |
| 12 | 0 | 0.00 |
| **Total** | **3507** | |

**Supplementary Table 3**. Number of strategies perceived as effective by lonely participants in the BBC dataset.

| **Number of strategies** | **BBC** | |
| --- | --- | --- |
|  | **N** | **%** |
| 0 | 1976 | 13.31 |
| 1 | 360 | 2.42 |
| 2 | 454 | 3.06 |
| 3 | 2524 | 17.00 |
| 4 | 1247 | 8.40 |
| 5 | 1286 | 8.66 |
| 6 | 1240 | 8.35 |
| 7 | 1144 | 7.71 |
| 8 | 1077 | 7.25 |
| 9 | 846 | 5.70 |
| 10 | 669 | 4.51 |
| 11 | 534 | 3.60 |
| 12 | 410 | 2.76 |
| 13 | 332 | 2.24 |
| 14 | 233 | 1.57 |
| 15 | 149 | 1.00 |
| 16 | 139 | 0.94 |
| 17 | 82 | 0.55 |
| 18 | 64 | 0.43 |
| 19 | 35 | 0.24 |
| 20 | 27 | 0.18 |
| 21 | 19 | 0.13 |
| **Total** | **14847** | |

**Supplementary Table 4**. Binary logistic regression analyses of the associations between use of strategies in the EU loneliness survey and Gender, Age Category and Region predictors.

| **Variable** |  | ***B*** | ***SE*** | **OR** | **95% CIs** | | ***p*-value** | **Direction of significant prediction** | **Nagelkerke R^2^** |
| --- | --- | --- | --- | --- | --- | --- | --- | --- | --- |
|  |  |  |  |  | **Lower** | **Upper** |  |  |  |
| Seeing friends, family members, or other loved ones | | | | | | | | | |
| Gender | Male |  |  | 1.000 |  |  | .177 |  |  |
|  | Female | 0.137 | 0.074 | 1.147 | 0.992 | 1.327 | .065 |  |  |
|  | Other | -0.006 | 0.393 | 0.994 | 0.460 | 2.147 | .987 |  |  |
| Age Category | Adults |  |  | 1.000 |  |  | <.001 |  |  |
|  | Young adults | 0.406 | 0.098 | 1.501 | 1.239 | 1.817 | <.001 | Young adults > adults |  |
|  | Older adults | 0.012 | 0.121 | 1.012 | 0.798 | 1.284 | .921 |  |  |
| Region | Western |  |  | 1.000 |  |  | <.001 |  |  |
|  | Northern | 0.443 | 0.105 | 1.558 | 1.268 | 1.915 | <.001 | Northern > Western |  |
|  | Southern | 0.497 | 0.109 | 1.645 | 1.328 | 2.036 | <.001 | Southern > Western |  |
|  | Central and Eastern | 0.453 | 0.101 | 1.573 | 1.291 | 1.918 | <.001 | Central and Eastern > Western |  |
|  |  |  |  |  |  |  |  |  | .021 |
| Doing sports alone | | | | | | | | | |
| Gender | Male |  |  | 1.000 |  |  | .003 |  |  |
|  | Female | -0.276 | 0.085 | 0.759 | 0.643 | 0.895 | .001 |  |  |
|  | Other | -0.611 | 0.496 | 0.543 | 0.206 | 1.435 | .218 |  |  |
| Age Category | Adults |  |  | 1.000 |  |  | <.001 |  |  |
|  | Young adults | 0.411 | 0.109 | 1.508 | 1.219 | 1.865 | <.001 | Young adults > adults |  |
|  | Older adults | -0.249 | 0.150 | 0.780 | 0.581 | 1.046 | .097 |  |  |
| Region | Western |  |  | 1.000 |  |  | .165 |  |  |
|  | Northern | 0.075 | 0.119 | 1.078 | 0.854 | 1.361 | .529 |  |  |
|  | Southern | 0.250 | 0.121 | 1.284 | 1.013 | 1.628 | .039 |  |  |
|  | Central and Eastern | 0.025 | 0.115 | 1.025 | 0.818 | 1.285 | .828 |  |  |
|  |  |  |  |  |  |  |  |  | .015 |
| Doing sports with others | | | | | | | | | |
| Gender | Male |  |  | 1.000 |  |  | <.001 |  |  |
|  | Female | -0.711 | 0.112 | 0.491 | 0.394 | 0.612 | <.001 | Female < male |  |
|  | Other | -0.342 | 0.546 | 0.711 | 0.244 | 2.071 | .531 |  |  |
| Age Category | Adults |  |  | 1.000 |  |  | <.001 |  |  |
|  | Young adults | 0.451 | 0.136 | 1.570 | 1.203 | 2.049 | <.001 | Young adults > adults |  |
|  | Older adults | -0.738 | 0.239 | 0.478 | 0.299 | 0.764 | .002 |  |  |
| Region | Western |  |  | 1.000 |  |  | .704 |  |  |
|  | Northern | -0.004 | 0.152 | 0.996 | 0.739 | 1.341 | .978 |  |  |
|  | Southern | 0.047 | 0.157 | 1.048 | 0.770 | 1.426 | .766 |  |  |
|  | Central and Eastern | -0.130 | 0.150 | 0.878 | 0.655 | 1.178 | .386 |  |  |
|  |  |  |  |  |  |  |  |  | .038 |
| Looked for self-help from books or online or called a support hotline | | | | | | | | | |
| Gender | Male |  |  | 1.000 |  |  | .050 |  |  |
|  | Female | 0.180 | 0.103 | 1.197 | 0.978 | 1.464 | .081 |  |  |
|  | Other | 0.822 | 0.423 | 2.276 | 0.994 | 5.211 | .052 |  |  |
| Age Category | Adults |  |  | 1.000 |  |  | .001 |  |  |
|  | Young adults | 0.391 | 0.126 | 1.478 | 1.154 | 1.894 | .002 |  |  |
|  | Older adults | -0.264 | 0.183 | 0.768 | 0.536 | 1.100 | .150 |  |  |
| Region | Western |  |  | 1.000 |  |  | .081 |  |  |
|  | Northern | 0.244 | 0.146 | 1.277 | 0.959 | 1.700 | .094 |  |  |
|  | Southern | 0.316 | 0.150 | 1.372 | 1.023 | 1.841 | .035 |  |  |
|  | Central and Eastern | 0.331 | 0.138 | 1.392 | 1.061 | 1.826 | .017 |  |  |
|  |  |  |  |  |  |  |  |  | .014 |
| Sought professional help by a therapist | | | | | | | | | |
| Gender | Male |  |  | 1.000 |  |  | .108 |  |  |
|  | Female | 0.156 | 0.104 | 1.169 | 0.954 | 1.433 | .132 |  |  |
|  | Other | 0.715 | 0.426 | 2.044 | 0.887 | 4.710 | .093 |  |  |
| Age Category | Adults |  |  | 1.000 |  |  | <.001 |  |  |
|  | Young adults | 0.531 | 0.123 | 1.700 | 1.335 | 2.165 | <.001 | Young adults > adults |  |
|  | Older adults | -0.733 | 0.220 | 0.480 | 0.312 | 0.739 | <.001 | Older adults < adults |  |
| Region | Western |  |  | 1.000 |  |  | .008 |  |  |
|  | Northern | -0.251 | 0.138 | 0.778 | 0.594 | 1.018 | .068 |  |  |
|  | Southern | -0.269 | 0.145 | 0.764 | 0.576 | 1.015 | .063 |  |  |
|  | Central and Eastern | -0.464 | 0.137 | 0.629 | 0.481 | 0.822 | <.001 |  |  |
|  |  |  |  |  |  |  |  |  | .028 |
| Contacted a specialized charity, association or non-governmental organization | | | | | | | | | |
| Gender | Male |  |  | 1.000 |  |  | <.001 |  |  |
|  | Female | -0.726 | 0.159 | 0.484 | 0.354 | 0.661 | <.001 | Female < male |  |
|  | Other | 0.706 | 0.556 | 2.026 | 0.681 | 6.023 | .204 |  |  |
| Age Category | Adults |  |  | 1.000 |  |  | .036 |  |  |
|  | Young adults | -0.352 | 0.233 | 0.703 | 0.445 | 1.111 | .131 |  |  |
|  | Older adults | -0.713 | 0.319 | 0.490 | 0.262 | 0.917 | .026 |  |  |
| Region | Western |  |  | 1.000 |  |  | <.001 |  |  |
|  | Northern | -0.565 | 0.200 | 0.568 | 0.384 | 0.841 | .005 |  |  |
|  | Southern | -0.689 | 0.219 | 0.502 | 0.327 | 0.772 | .002 |  |  |
|  | Central and Eastern | -0.977 | 0.216 | 0.377 | 0.247 | 0.575 | <.001 | Central and Eastern < Western |  |
|  |  |  |  |  |  |  |  |  | .049 |
| Contacted a church or religious organization | | | | | | | | | |
| Gender | Male |  |  | 1.000 |  |  | <.001 |  |  |
|  | Female | -0.546 | 0.147 | 0.579 | 0.434 | 0.773 | <.001 | Female < male |  |
|  | Other | 1.156 | 0.473 | 3.179 | 1.258 | 8.034 | .014 |  |  |
| Age Category | Adults |  |  | 1.000 |  |  | .358 |  |  |
|  | Young adults | -0.187 | 0.209 | 0.830 | 0.551 | 1.249 | .370 |  |  |
|  | Older adults | -0.320 | 0.262 | 0.726 | 0.434 | 1.214 | .223 |  |  |
| Region | Western |  |  | 1.000 |  |  | .036 |  |  |
|  | Northern | -0.099 | 0.190 | 0.906 | 0.624 | 1.313 | .601 |  |  |
|  | Southern | -0.670 | 0.234 | 0.511 | 0.323 | 0.809 | .004 |  |  |
|  | Central and Eastern | -0.167 | 0.185 | 0.846 | 0.589 | 1.216 | .367 |  |  |
|  |  |  |  |  |  |  |  |  | .026 |
| Joined a club or group | | | | | | | | | |
| Gender | Male |  |  | 1.000 |  |  | <.001 |  |  |
|  | Female | -0.585 | 0.121 | 0.557 | 0.440 | 0.706 | <.001 | Female < male |  |
|  | Other | 0.009 | 0.549 | 1.009 | 0.344 | 2.961 | .987 |  |  |
| Age Category | Adults |  |  | 1.000 |  |  | .348 |  |  |
|  | Young adults | 0.084 | 0.161 | 1.087 | 0.793 | 1.491 | .603 |  |  |
|  | Older adults | -0.271 | 0.213 | 0.763 | 0.503 | 1.158 | .203 |  |  |
| Region | Western |  |  | 1.000 |  |  | <.001 |  |  |
|  | Northern | -0.521 | 0.155 | 0.594 | 0.438 | 0.805 | <.001 | Northern < Western |  |
|  | Southern | -0.651 | 0.170 | 0.522 | 0.374 | 0.728 | <.001 | Southern < Western |  |
|  | Central and Eastern | -0.893 | 0.164 | 0.409 | 0.297 | 0.564 | <.001 | Central and Eastern < Western |  |
|  |  |  |  |  |  |  |  |  | .039 |
| Volunteered | | | | | | | | | |
| Gender | Male |  |  | 1.000 |  |  | .075 |  |  |
|  | Female | -0.296 | 0.131 | 0.744 | 0.576 | 0.961 | .024 |  |  |
|  | Other | -0.300 | 0.740 | 0.741 | 0.174 | 3.161 | .686 |  |  |
| Age Category | Adults |  |  | 1.000 |  |  | .780 |  |  |
|  | Young adults | -0.119 | 0.187 | 0.888 | 0.615 | 1.280 | .523 |  |  |
|  | Older adults | -0.084 | 0.217 | 0.919 | 0.601 | 1.407 | .699 |  |  |
| Region | Western |  |  | 1.000 |  |  | .060 |  |  |
|  | Northern | -0.170 | 0.177 | 0.843 | 0.596 | 1.193 | .335 |  |  |
|  | Southern | -0.010 | 0.178 | 0.990 | 0.698 | 1.403 | .953 |  |  |
|  | Central and Eastern | -0.450 | 0.181 | 0.638 | 0.447 | 0.910 | .013 |  |  |
|  |  |  |  |  |  |  |  |  | .010 |
| Used more social media | | | | | | | | | |
| Gender | Male |  |  | 1.000 |  |  | .006 |  |  |
|  | Female | 0.241 | 0.077 | 1.273 | 1.095 | 1.480 | .002 |  |  |
|  | Other | -0.150 | 0.420 | 0.861 | 0.378 | 1.961 | .721 |  |  |
| Age Category | Adults |  |  | 1.000 |  |  | <.001 |  |  |
|  | Young adults | 0.472 | 0.100 | 1.603 | 1.319 | 1.948 | <.001 | Young adults > adults |  |
|  | Older adults | 0.048 | 0.125 | 1.049 | 0.821 | 1.341 | .702 |  |  |
| Region | Western |  |  | 1.000 |  |  | <.001 |  |  |
|  | Northern | 0.391 | 0.109 | 1.479 | 1.194 | 1.831 | <.001 | Northern > Western |  |
|  | Southern | 0.628 | 0.111 | 1.873 | 1.506 | 2.329 | <.001 | Southern > Western |  |
|  | Central and Eastern | 0.341 | 0.105 | 1.407 | 1.144 | 1.730 | .001 |  |  |
|  |  |  |  |  |  |  |  |  | .027 |
| Took time for myself | | | | | | | | | |
| Gender | Male |  |  | 1.000 |  |  | .072 |  |  |
|  | Female | 0.172 | 0.076 | 1.187 | 1.022 | 1.378 | .024 |  |  |
|  | Other | 0.269 | 0.384 | 1.309 | 0.617 | 2.779 | .483 |  |  |
| Age Category | Adults |  |  | 1.000 |  |  | <.001 |  |  |
|  | Young adults | 0.414 | 0.099 | 1.513 | 1.246 | 1.837 | <.001 | Young adults > adults |  |
|  | Older adults | -0.096 | 0.127 | 0.908 | 0.708 | 1.165 | .448 |  |  |
| Region | Western |  |  | 1.000 |  |  | .003 |  |  |
|  | Northern | 0.256 | 0.107 | 1.291 | 1.046 | 1.594 | .017 |  |  |
|  | Southern | 0.375 | 0.111 | 1.456 | 1.172 | 1.808 | <.001 |  |  |
|  | Central and Eastern | 0.312 | 0.103 | 1.366 | 1.118 | 1.670 | .002 |  |  |
|  |  |  |  |  |  |  |  |  | .016 |
| Wanted to do something but did not know what to do | | | | | | | | | |
| Gender | Male |  |  | 1.000 |  |  | <.001 |  |  |
|  | Female | 0.412 | 0.091 | 1.510 | 1.263 | 1.806 | <.001 | Female > male |  |
|  | Other | -0.407 | 0.614 | 0.666 | 0.200 | 2.217 | .508 |  |  |
| Age Category | Adults |  |  | 1.000 |  |  | .159 |  |  |
|  | Young adults | -0.140 | 0.127 | 0.870 | 0.678 | 1.115 | .272 |  |  |
|  | Older adults | 0.191 | 0.138 | 1.211 | 0.924 | 1.587 | .166 |  |  |
| Region | Western |  |  | 1.000 |  |  | .068 |  |  |
|  | Northern | 0.328 | 0.125 | 1.388 | 1.087 | 1.773 | .183 |  |  |
|  | Southern | 0.177 | 0.133 | 1.194 | 0.920 | 1.549 | .183 |  |  |
|  | Central and Eastern | 0.217 | 0.122 | 1.242 | 0.978 | 1.576 | .075 |  |  |
|  |  |  |  |  |  |  |  |  | .016 |
| Other, not listed above | | | | | | | | | |
| Gender | Male |  |  | 1.000 |  |  | .040 |  |  |
|  | Female | -0.462 | 0.192 | 0.630 | 0.433 | 0.917 | .016 |  |  |
|  | Other | 0.383 | 0.750 | 1.467 | 0.337 | 6.378 | .609 |  |  |
| Age Category | Adults |  |  | 1.000 |  |  | .036 |  |  |
|  | Young adults | 0.463 | 0.238 | 1.589 | 0.996 | 2.535 | .052 |  |  |
|  | Older adults | 0.561 | 0.270 | 1.752 | 1.033 | 2.972 | .037 |  |  |
| Region | Western |  |  | 1.000 |  |  | .179 |  |  |
|  | Northern | 0.588 | 0.279 | 1.800 | 1.043 | 3.106 | .035 |  |  |
|  | Southern | 0.510 | 0.292 | 1.666 | 0.940 | 2.953 | .081 |  |  |
|  | Central and Eastern | 0.436 | 0.278 | 1.546 | 0.896 | 2.668 | .117 |  |  |
|  |  |  |  |  |  |  |  |  | .020 |
| None of the above | | | | | | | | | |
| Gender | Male |  |  | 1.000 |  |  | .729 |  |  |
|  | Female | 0.083 | 0.124 | 1.086 | 0.852 | 1.386 | .504 |  |  |
|  | Other | 0.314 | 0.622 | 1.369 | 0.405 | 4.633 | .613 |  |  |
| Age Category | Adults |  |  | 1.000 |  |  | <.001 |  |  |
|  | Young adults | -0.489 | 0.202 | 0.613 | 0.413 | 0.911 | .015 |  |  |
|  | Older adults | 0.495 | 0.170 | 1.641 | 1.177 | 2.288 | .004 |  |  |
| Region | Western |  |  | 1.000 |  |  | .002 |  |  |
|  | Northern | -0.555 | 0.172 | 0.574 | 0.410 | 0.804 | .001 |  |  |
|  | Southern | -0.389 | 0.172 | 0.678 | 0.484 | 0.950 | .024 |  |  |
|  | Central and Eastern | -0.475 | 0.159 | 0.622 | 0.455 | 0.850 | .003 |  |  |
|  |  |  |  |  |  |  |  |  | .021 |

**Notes**. *B* = estimated regression coefficient, *SE* = standard error, OR = odds ratio, 95% CIs = 95% confidence intervals. The first level of each predictor variable is the reference category. ORs > 1 indicate that level of the predictor is more likely to use the strategy than the reference and ORs < 1 indicate that level of the predictor is less likely to use the strategy than the reference. *p* < .001 is significant.

**Supplementary Table 5**. Binary logistic regression analyses of the associations between perceived effectiveness of strategies in the BBC loneliness survey and Gender, Age category and Income predictors.

| **Variable** |  | ***B*** | ***SE*** | **OR** | **95% CIs** | | ***p*-value** | **Direction of significant predication** | **Nagelkerke R^2^** |
| --- | --- | --- | --- | --- | --- | --- | --- | --- | --- |
|  |  |  |  |  | **Lower** | **Upper** |  |  |  |
| Join a club | | | | | | | | | |
| Gender | Male |  |  | 1.000 |  |  | .002 |  |  |
|  | Female | 0.124 | 0.036 | 1.132 | 1.056 | 1.215 | <.001 |  |  |
|  | Other | -0.078 | 0.219 | 0.925 | 0.602 | 1.421 | .722 |  |  |
| Age Category | Adults |  |  | 1.000 |  |  | <.001 |  |  |
|  | Young adults | -0.073 | 0.062 | 0.930 | 0.824 | 1.049 | .238 |  |  |
|  | Older adults | 0.135 | 0.039 | 1.145 | 1.060 | 1.237 | <.001 | Older adults > adults |  |
| Income | Poorly |  |  | 1.000 |  |  | <.001 |  |  |
|  | Fairly | 0.184 | 0.041 | 1.202 | 1.109 | 1.302 | <.001 | Fairly > poorly |  |
|  | Very | 0.341 | 0.049 | 1.406 | 1.278 | 1.547 | <.001 | Very > poorly |  |
|  |  |  |  |  |  |  |  |  | .008 |
| Find activities to distract you when on your own | | | | | | | | | |
| Gender | Male |  |  | 1.000 |  |  | <.001 |  |  |
|  | Female | 0.300 | 0.035 | 1.349 | 1.259 | 1.446 | <.001 | Female > male |  |
|  | Other | 0.118 | 0.211 | 1.125 | 0.743 | 1.703 | .577 |  |  |
| Age Category | Adults |  |  | 1.000 |  |  | <.001 |  |  |
|  | Young adults | 0.200 | 0.062 | 1.221 | 1.082 | 1.378 | .001 |  |  |
|  | Older adults | 0.146 | 0.040 | 1.157 | 1.071 | 1.251 | <.001 | Older adults > adults |  |
| Income | Poorly |  |  | 1.000 |  |  | .197 |  |  |
|  | Fairly | 0.073 | 0.040 | 1.075 | 0.994 | 1.163 | .071 |  |  |
|  | Very | 0.047 | 0.049 | 1.048 | 0.953 | 1.152 | .336 |  |  |
|  |  |  |  |  |  |  |  |  | .009 |
| Dedicate time to work, study or hobbies | | | | | | | | | |
| Gender | Male |  |  | 1.000 |  |  | <.001 |  |  |
|  | Female | 0.210 | 0.035 | 1.233 | 1.152 | 1.321 | <.001 | Female > male |  |
|  | Other | 0.165 | 0.210 | 1.179 | 0.782 | 1.779 | .431 |  |  |
| Age Category | Adults |  |  | 1.000 |  |  | .007 |  |  |
|  | Young adults | 0.122 | 0.060 | 1.130 | 1.003 | 1.272 | .044 |  |  |
|  | Older adults | -0.077 | 0.039 | 0.926 | 0.858 | 1.000 | .049 |  |  |
| Income | Poorly |  |  | 1.000 |  |  | .131 |  |  |
|  | Fairly | 0.064 | 0.040 | 1.067 | 0.987 | 1.153 | .104 |  |  |
|  | Very | 0.090 | 0.048 | 1.094 | 0.996 | 1.202 | .060 |  |  |
|  |  |  |  |  |  |  |  |  | .005 |
| Look for a new job | | | | | | | | | |
| Gender | Male |  |  | 1.000 |  |  | .084 |  |  |
|  | Female | 0.102 | 0.053 | 1.107 | 0.998 | 1.228 | .054 |  |  |
|  | Other | -0.308 | 0.339 | 0.735 | 0.378 | 1.428 | .363 |  |  |
| Age Category | Adults |  |  | 1.000 |  |  | <.001 |  |  |
|  | Young adults | -0.079 | 0.087 | 0.924 | 0.780 | 1.096 | .364 |  |  |
|  | Older adults | -0.861 | 0.071 | 0.423 | 0.368 | 0.486 | <.001 | Older adults < adults |  |
| Income | Poorly |  |  | 1.000 |  |  | <.001 |  |  |
|  | Fairly | -0.346 | 0.056 | 0.708 | 0.634 | 0.790 | <.001 | Fairly < poorly |  |
|  | Very | -0.485 | 0.073 | 0.616 | 0.534 | 0.710 | <.001 | Very < poorly |  |
|  |  |  |  |  |  |  |  |  | .032 |
| Introduce yourself to neighbors | | | | | | | | | |
| Gender | Male |  |  | 1.000 |  |  | <.001 |  |  |
|  | Female | 0.345 | 0.062 | 1.412 | 1.250 | 1.596 | <.001 | Female > male |  |
|  | Other | 0.063 | 0.398 | 1.065 | 0.489 | 2.321 | .874 |  |  |
| Age Category | Adults |  |  | 1.000 |  |  | <.001 |  |  |
|  | Young adults | -0.167 | 0.111 | 0.846 | 0.681 | 1.052 | .132 |  |  |
|  | Older adults | 0.223 | 0.063 | 1.249 | 1.104 | 1.414 | <.001 | Older adults > adults |  |
| Income | Poorly |  |  | 1.000 |  |  | .076 |  |  |
|  | Fairly | 0.082 | 0.069 | 1.085 | 0.947 | 1.243 | .239 |  |  |
|  | Very | 0.183 | 0.081 | 1.201 | 1.025 | 1.407 | .024 |  |  |
|  |  |  |  |  |  |  |  |  | .009 |
| Move to a new area | | | | | | | | | |
| Gender | Male |  |  | 1.000 |  |  | .266 |  |  |
|  | Female | -0.090 | 0.058 | 0.914 | 0.816 | 1.023 | .118 |  |  |
|  | Other | -0.214 | 0.355 | 0.807 | 0.403 | 1.619 | .547 |  |  |
| Age Category | Adults |  |  | 1.000 |  |  | .124 |  |  |
|  | Young adults | 0.161 | 0.096 | 1.175 | 0.974 | 1.417 | .092 |  |  |
|  | Older adults | -0.053 | 0.066 | 0.948 | 0.833 | 1.079 | .420 |  |  |
| Income | Poorly |  |  | 1.000 |  |  | <.001 |  |  |
|  | Fairly | -0.329 | 0.063 | 0.719 | 0.636 | 0.813 | <.001 | Fairly < poorly |  |
|  | Very | -0.485 | 0.081 | 0.616 | 0.526 | 0.722 | <.001 | Very < poorly |  |
|  |  |  |  |  |  |  |  |  | .007 |
| Re-engage with your church, mosque or equivalent | | | | | | | | | |
| Gender | Male |  |  | 1.000 |  |  | <.001 |  |  |
|  | Female | 0.383 | 0.053 | 1.466 | 1.321 | 1.628 | <.001 | Female > male |  |
|  | Other | 0.227 | 0.313 | 1.254 | 0.679 | 2.318 | .470 |  |  |
| Age Category | Adults |  |  | 1.000 |  |  | .092 |  |  |
|  | Young adults | -0.171 | 0.092 | 0.843 | 0.704 | 1.010 | .064 |  |  |
|  | Older adults | 0.045 | 0.056 | 1.046 | 0.938 | 1.166 | .421 |  |  |
| Income | Poorly |  |  | 1.000 |  |  | .738 |  |  |
|  | Fairly | -0.003 | 0.058 | 0.997 | 0.890 | 1.117 | .962 |  |  |
|  | Very | 0.043 | 0.069 | 1.044 | 0.912 | 1.196 | .530 |  |  |
|  |  |  |  |  |  |  |  |  | .008 |
| Tell someone | | | | | | | | | |
| Gender | Male |  |  | 1.000 |  |  | <.001 |  |  |
|  | Female | 0.276 | 0.043 | 1.318 | 1.213 | 1.433 | <.001 | Female > male |  |
|  | Other | 0.427 | 0.226 | 1.533 | 0.985 | 2.387 | .058 |  |  |
| Age Category | Adults |  |  | 1.000 |  |  | <.001 |  |  |
|  | Young adults | 0.632 | 0.063 | 1.881 | 1.661 | 2.130 | <.001 | Young adults > adults |  |
|  | Older adults | -0.392 | 0.049 | 0.676 | 0.613 | 0.745 | <.001 | Older adults < adults |  |
| Income | Poorly |  |  | 1.000 |  |  | .879 |  |  |
|  | Fairly | -0.009 | 0.047 | 0.991 | 0.903 | 1.087 | .843 |  |  |
|  | Very | 0.016 | 0.057 | 1.017 | 0.909 | 1.136 | .772 |  |  |
|  |  |  |  |  |  |  |  |  | .025 |
| Talk to family and friends about your feelings | | | | | | | | | |
| Gender | Male |  |  | 1.000 |  |  | <.001 |  |  |
|  | Female | 0.300 | 0.039 | 1.349 | 1.249 | 1.458 | <.001 | Female > male |  |
|  | Other | 0.287 | 0.221 | 1.332 | 0.864 | 2.053 | .194 |  |  |
| Age Category | Adults |  |  | 1.000 |  |  | <.001 |  |  |
|  | Young adults | 0.636 | 0.061 | 1.889 | 1.675 | 2.129 | <.001 | Young adults > adults |  |
|  | Older adults | -0.311 | 0.045 | 0.733 | 0.671 | 0.800 | <.001 | Older adults < adults |  |
| Income | Poorly |  |  | 1.000 |  |  | .123 |  |  |
|  | Fairly | 0.074 | 0.044 | 1.076 | 0.987 | 1.173 | .095 |  |  |
|  | Very | 0.101 | 0.053 | 1.106 | 0.997 | 1.227 | .057 |  |  |
|  |  |  |  |  |  |  |  |  | .025 |
| Invite people to be friends without fearing rejection | | | | | | | | | |
| Gender | Male |  |  | 1.000 |  |  | <.001 |  |  |
|  | Female | 0.517 | 0.047 | 1.678 | 1.530 | 1.840 | <.001 | Female > male |  |
|  | Other | 0.633 | 0.242 | 1.883 | 1.173 | 3.023 | .009 |  |  |
| Age Category | Adults |  |  | 1.000 |  |  | <.001 |  |  |
|  | Young adults | 0.259 | 0.071 | 1.295 | 1.127 | 1.488 | <.001 | Young adults > adults |  |
|  | Older adults | -0.234 | 0.051 | 0.791 | 0.716 | 0.875 | <.001 | Older adults < adults |  |
| Income | Poorly |  |  | 1.000 |  |  | <.001 |  |  |
|  | Fairly | 0.159 | 0.052 | 1.172 | 1.059 | 1.298 | .002 |  |  |
|  | Very | 0.346 | 0.060 | 1.413 | 1.255 | 1.591 | <.001 | Very > poorly |  |
|  |  |  |  |  |  |  |  |  | .022 |
| Look for the good in everyone you meet | | | | | | | | | |
| Gender | Male |  |  | 1.000 |  |  | <.001 |  |  |
|  | Female | 0.298 | 0.040 | 1.347 | 1.245 | 1.458 | <.001 | Female > male |  |
|  | Other | 0.271 | 0.234 | 1.312 | 0.830 | 2.074 | .246 |  |  |
| Age Category | Adults |  |  | 1.000 |  |  | <.001 |  |  |
|  | Young adults | 0.013 | 0.068 | 1.013 | 0.886 | 1.158 | 0.854 |  |  |
|  | Older adults | 0.227 | 0.043 | 1.254 | 1.154 | 1.364 | <.001 | Older adults > adults |  |
| Income | Poorly |  |  | 1.000 |  |  | .438 |  |  |
|  | Fairly | 0.034 | 0.045 | 1.035 | 0.947 | 1.130 | .450 |  |  |
|  | Very | 0.069 | 0.054 | 1.071 | 0.964 | 1.191 | .199 |  |  |
|  |  |  |  |  |  |  |  |  | .009 |
| Start a conversation with anyone you interact with | | | | | | | | | |
| Gender | Male |  |  | 1.000 |  |  | <.001 |  |  |
|  | Female | 0.484 | 0.039 | 1.622 | 1.502 | 1.751 | <.001 | Female > male |  |
|  | Other | -0.024 | 0.257 | 0.977 | 0.590 | 1.617 | .927 |  |  |
| Age Category | Adults |  |  | 1.000 |  |  | <.001 |  |  |
|  | Young adults | -0.494 | 0.074 | 0.610 | 0.528 | 0.705 | <.001 | Young adults < adults |  |
|  | Older adults | 0.653 | 0.040 | 1.922 | 1.776 | 2.080 | <.001 | Older adults > adults |  |
| Income | Poorly |  |  | 1.000 |  |  | .125 |  |  |
|  | Fairly | -0.068 | 0.043 | 0.934 | 0.858 | 1.017 | .115 |  |  |
|  | Very | 0.012 | 0.052 | 1.012 | 0.915 | 1.120 | .813 |  |  |
|  |  |  |  |  |  |  |  |  | .051 |
| Seek counselling | | | | | | | | | |
| Gender | Male |  |  | 1.000 |  |  | <.001 |  |  |
|  | Female | 0.205 | 0.039 | 1.227 | 1.137 | 1.325 | <.001 | Female > male |  |
|  | Other | 0.430 | 0.217 | 1.538 | 1.005 | 2.352 | .047 |  |  |
| Age Category | Adults |  |  | 1.000 |  |  | <.001 |  |  |
|  | Young adults | -0.007 | 0.065 | 0.993 | 0.874 | 1.129 | .916 |  |  |
|  | Older adults | -0.370 | 0.045 | 0.691 | 0.633 | 0.754 | <.001 | Older adults < adults |  |
| Income | Poorly |  |  | 1.000 |  |  | <.001 |  |  |
|  | Fairly | -0.176 | 0.043 | 0.838 | 0.770 | 0.912 | <.001 | Fairly < poorly |  |
|  | Very | -0.222 | 0.053 | 0.801 | 0.722 | 0.888 | <.001 | Very < poorly |  |
|  |  |  |  |  |  |  |  |  | .013 |
| Find new non social activities and pastimes | | | | | | | | | |
| Gender | Male |  |  | 1.000 |  |  | .002 |  |  |
|  | Female | 0.149 | 0.044 | 1.161 | 1.066 | 1.265 | <.001 |  |  |
|  | Other | -0.040 | 0.266 | 0.961 | 0.571 | 1.617 | .881 |  |  |
| Age Category | Adults |  |  | 1.000 |  |  | <.001 | Unclear |  |
|  | Young adults | 0.231 | 0.071 | 1.260 | 1.096 | 1.449 | .001 |  |  |
|  | Older adults | 0.146 | 0.047 | 1.157 | 1.055 | 1.269 | .002 |  |  |
| Income | Poorly |  |  | 1.000 |  |  | .224 |  |  |
|  | Fairly | -0.032 | 0.049 | 0.969 | 0.880 | 1.067 | .519 |  |  |
|  | Very | 0.058 | 0.058 | 1.060 | 0.945 | 1.188 | .318 |  |  |
|  |  |  |  |  |  |  |  |  | .004 |
| Find new friends | | | | | | | | | |
| Gender | Male |  |  | 1.000 |  |  | .507 |  |  |
|  | Female | 0.045 | 0.043 | 1.046 | 0.961 | 1.137 | .301 |  |  |
|  | Other | 0.158 | 0.244 | 1.171 | 0.726 | 1.889 | .516 |  |  |
| Age Category | Adults |  |  | 1.000 |  |  | <.001 |  |  |
|  | Young adults | 0.274 | 0.069 | 1.315 | 1.148 | 1.506 | <.001 | Young adults > adults |  |
|  | Older adults | -0.028 | 0.048 | 0.972 | 0.885 | 1.068 | .556 |  |  |
| Income | Poorly |  |  | 1.000 |  |  | .068 |  |  |
|  | Fairly | 0.050 | 0.049 | 1.051 | 0.955 | 1.158 | .308 |  |  |
|  | Very | 0.134 | 0.058 | 1.144 | 1.020 | 1.282 | .021 |  |  |
|  |  |  |  |  |  |  |  |  | .003 |
| Use the internet for support | | | | | | | | | |
| Gender | Male |  |  | 1.000 |  |  | <.001 |  |  |
|  | Female | 0.191 | 0.042 | 1.211 | 1.115 | 1.315 | <.001 | Female > male |  |
|  | Other | 1.196 | 0.212 | 3.307 | 2.182 | 5.011 | <.001 | Other > male |  |
| Age Category | Adults |  |  | 1.000 |  |  | <.001 |  |  |
|  | Young adults | 0.579 | 0.064 | 1.785 | 1.573 | 2.025 | <.001 | Young adults > adults |  |
|  | Older adults | -0.108 | 0.047 | 0.898 | 0.818 | 0.985 | .023 |  |  |
| Income | Poorly |  |  | 1.000 |  |  | <.001 |  |  |
|  | Fairly | -0.186 | 0.046 | 0.830 | 0.759 | 0.908 | <.001 | Fairly < poorly |  |
|  | Very | -0.421 | 0.058 | 0.656 | 0.586 | 0.735 | <.001 | Very < poorly |  |
|  |  |  |  |  |  |  |  |  | .021 |
| Wait for the feeling to pass | | | | | | | | | |
| Gender | Male |  |  | 1.000 |  |  | .032 |  |  |
|  | Female | -0.093 | 0.037 | 0.911 | 0.847 | 0.980 | .012 |  |  |
|  | Other | 0.113 | 0.217 | 1.120 | 0.731 | 1.715 | .603 |  |  |
| Age Category | Adults |  |  | 1.000 |  |  | .858 |  |  |
|  | Young adults | -0.030 | 0.064 | 0.971 | 0.856 | 1.101 | .645 |  |  |
|  | Older adults | -0.016 | 0.041 | 0.984 | 0.907 | 1.067 | .699 |  |  |
| Income | Poorly |  |  | 1.000 |  |  | .078 |  |  |
|  | Fairly | 0.083 | 0.042 | 1.087 | 1.000 | 1.181 | .050 |  |  |
|  | Very | 0.102 | 0.051 | 1.108 | 1.002 | 1.224 | .045 |  |  |
|  |  |  |  |  |  |  |  |  | .001 |
| Give yourself time to think about why | | | | | | | | | |
| Gender | Male |  |  | 1.000 |  |  | <.001 |  |  |
|  | Female | 0.281 | 0.042 | 1.324 | 1.219 | 1.439 | <.001 | Female > male |  |
|  | Other | 0.551 | 0.222 | 1.735 | 1.123 | 2.680 | .013 |  |  |
| Age Category | Adults |  |  | 1.000 |  |  | <.001 |  |  |
|  | Young adults | 0.570 | 0.064 | 1.768 | 1.560 | 2.003 | <.001 | Young adults > adults |  |
|  | Older adults | -0.293 | 0.048 | 0.746 | 0.678 | 0.820 | <.001 | Older adults < adults |  |
| Income | Poorly |  |  | 1.000 |  |  | .253 |  |  |
|  | Fairly | 0.026 | 0.047 | 1.026 | 0.935 | 1.125 | .587 |  |  |
|  | Very | 0.091 | 0.056 | 1.095 | 0.981 | 1.223 | .107 |  |  |
|  |  |  |  |  |  |  |  |  | .020 |
| Don’t know what to do | | | | | | | | | |
| Gender | Male |  |  | 1.000 |  |  | <.001 |  |  |
|  | Female | -0.509 | 0.039 | 0.601 | 0.557 | 0.648 | <.001 | Female < male |  |
|  | Other | 0.121 | 0.215 | 1.129 | 0.741 | 1.719 | .573 |  |  |
| Age Category | Adults |  |  | 1.000 |  |  | <.001 |  |  |
|  | Young adults | 0.232 | 0.065 | 1.261 | 1.109 | 1.433 | <.001 | Young adults > adults |  |
|  | Older adults | -0.151 | 0.045 | 0.860 | 0.787 | 0.939 | <.001 | Older adults < adults |  |
| Income | Poorly |  |  | 1.000 |  |  | <.001 |  |  |
|  | Fairly | -0.234 | 0.044 | 0.792 | 0.727 | 0.863 | <.001 | Fairly < poorly |  |
|  | Very | -0.389 | 0.055 | 0.678 | 0.609 | 0.754 | <.001 | Very < poorly |  |
|  |  |  |  |  |  |  |  |  | .027 |
| Find new social activities and pastimes | | | | | | | | | |
| Gender | Male |  |  | 1.000 |  |  | <.001 |  |  |
|  | Female | 0.352 | 0.038 | 1.421 | 1.320 | 1.531 | <.001 | Female > male |  |
|  | Other | 0.140 | 0.229 | 1.151 | 0.735 | 1.801 | .539 |  |  |
| Age Category | Adults |  |  | 1.000 |  |  | .012 |  |  |
|  | Young adults | -0.128 | 0.065 | 0.880 | 0.774 | 1.000 | .049 |  |  |
|  | Older adults | 0.074 | 0.041 | 1.077 | 0.994 | 1.167 | .070 |  |  |
| Income | Poorly |  |  | 1.000 |  |  | <.001 |  |  |
|  | Fairly | 0.152 | 0.043 | 1.164 | 1.070 | 1.265 | <.001 | Fairly > poorly |  |
|  | Very | 0.270 | 0.051 | 1.311 | 1.186 | 1.448 | <.001 | Very > poorly |  |
|  |  |  |  |  |  |  |  |  | .013 |
| Change my thinking to be more positive | | | | | | | | | |
| Gender | Male |  |  | 1.000 |  |  | <.001 |  |  |
|  | Female | 0.342 | 0.037 | 1.408 | 1.310 | 1.513 | <.001 | Female > male |  |
|  | Other | -0.443 | 0.250 | 0.642 | 0.393 | 1.049 | .077 |  |  |
| Age Category | Adults |  |  | 1.000 |  |  | .002 |  |  |
|  | Young adults | -0.098 | 0.063 | 0.907 | 0.801 | 1.027 | .122 |  |  |
|  | Older adults | 0.110 | 0.040 | 1.117 | 1.032 | 1.207 | .006 |  |  |
| Income | Poorly |  |  | 1.000 |  |  | .049 |  |  |
|  | Fairly | 0.075 | 0.041 | 1.078 | 0.994 | 1.169 | .071 |  |  |
|  | Very | 0.118 | 0.050 | 1.125 | 1.021 | 1.240 | .018 |  |  |
|  |  |  |  |  |  |  |  |  | .011 |

**Notes**. *B* = estimated regression coefficient, *SE* = standard error, OR = odds ratio, 95% CIs = 95% confidence intervals. The first level of each predictor variable is the reference category. ORs > 1 indicate that level of the predictor is more likely to use the strategy than the reference and ORs < 1 indicate that level of the predictor is less likely to use the strategy than the reference. *p* < .001 is significant.
